# Probing apathy in children and adolescents with the Apathy Motivation Index–Child version

**DOI:** 10.3758/s13428-023-02184-4

**Published:** 2023-08-03

**Authors:** Samuel R.C. Hewitt, Johanna Habicht, Aislinn Bowler, Patricia L. Lockwood, Tobias U. Hauser

**Affiliations:** 1 Max Planck UCL Centre for Computational Psychiatry and Ageing Research, University College London, London, UK; 2 Wellcome Centre for Human Neuroimaging, University College London, 12 Queen Square, London, WC1N 3AR UK; 3 Centre for Brain and Cognitive Development, Birkbeck, University of London, London, WC1E 7HX UK; 4 Department of Experimental Psychology, University of Oxford, Oxford, UK; 5 Centre for Human Brain Health, School of Psychology, University of Birmingham, Birmingham, UK; 6 Institute for Mental Health, School of Psychology, University of Birmingham, Birmingham, UK; 7 Centre for Developmental Science, School of Psychology, University of Birmingham, Birmingham, UK; 8 Department of Psychiatry and Psychotherapy, Medical School and University Hospital, Eberhard Karls University of Tübingen, Tübingen, Germany

**Keywords:** Apathy, Motivation, Self-report, Development, Psychiatry, Mental health

## Abstract

Apathy is linked to mental health and altered neurocognitive functions such as learning and decision-making in healthy adults. Mental health problems typically begin to emerge during adolescence, yet little is known about how apathy develops due to an absence of quantitative measurements specific to young people. Here, we present and evaluate the Apathy Motivation Index–Child Version (AMI-CV) for children and adolescents. We show across two samples of young people (aged 8 to 17 years, total *N* = 191) tested in schools in the UK and on a smartphone app, that the AMI-CV is a short, psychometrically sound measure to assess levels of apathy and motivation in young people. Similar to adult versions, the AMI-CV captures three distinct apathy domains: Behavioural Activation, Social Motivation and Emotional Sensitivity. The AMI-CV showed excellent construct validity with an alternative measure of apathy and external validity replicating specific links with related mental health traits shown in adults. Our results provide a short measure of self-reported apathy in young people that enables research into apathy development. The AMI-CV can be used in conjunction with the adult version to investigate the impact of levels of apathy across the lifespan.

## Introduction

Motivation is an internal state associated with the willingness to initiate and maintain goal-directed behaviours (Chong et al., 2016). Motivation is critical to good physical and mental health because these require goal-directed behaviours such as maintaining a stable income, eating a balanced diet, doing exercise, socialising and regulating risky behaviour. In healthy adults, reduced trait motivation, also termed apathy, is composed of at least three dimensions covering engagement in behavioural, social and emotional domains (Ang et al., 2017). These are differentially linked to various other important traits and behaviours, such as depression and anhedonia (Ang et al., 2017), fatigue (Daumas et al., 2022), impulsivity (Petitet et al., 2021), empathy (Lockwood, Ang, et al., 2017a), prosocial behaviour (Lockwood, Hamonet, et al., 2017b), willingness to exert effort for reward (Daumas et al., 2022; Jurgelis et al., 2021), planning of future behaviour (Scholl et al., 2022) and learning (Hauser et al., 2017).

Adolescence may be a period of apathy development in some young people. This would be of particular clinical and scientific interest because high levels of apathy are a pervasive symptom of several common psychiatric disorders and the majority of these emerge during adolescence (Hauser et al., 2019; Paus et al., 2008). Decreases in goal-directed behaviour (behavioural activation) are cardinal features of major depression and negative symptoms of schizophrenia (Barch et al., 2016), while behavioural and social apathy are comorbid in some patients with anxiety disorders (Hellström et al., 2014). Apathy also occurs in disorders characterised by increased compulsive engagement such as obsessive compulsive–disorder (Raffard et al., 2020), attention-deficit/hyperactivity disorder in children (Torrente et al., 2011) and addiction in adults (Verdejo-García et al., 2006). In healthy children and adolescents, the relationships between levels of apathy and internalising (e.g., anxiety–depression) and externalising traits (e.g., impulsivity–compulsivity) are largely unknown.

Since adolescence is a period of accelerated biological, psychological and social change (Sawyer et al., 2018), we expect that levels of apathy during this complex developmental process may predict important outcomes (e.g., biological, psychosocial, educational). However, very little is known about how apathy varies and develops over time because there is no established method for the assessment of apathy in children and adolescents.

In this study, we developed the Apathy Motivation Index–Child Version (AMI-CV), a brief, self-report questionnaire for children and adolescents to assess levels of apathy. We adapted the Apathy-Motivation Index for adults (Ang et al., 2017) to develop an age-appropriate version, and validated this using data from two independent samples of children in schools in the UK and on a smartphone app. The AMI-CV replicates the factor structure of the AMI in adults. We also found that levels of behavioural, social and emotional apathy in children and adolescents replicated relationships with several mental health traits in adults, demonstrating the ecological and external validity of the AMI-CV.

## Methods

### Participants

Two samples of children and adolescents were recruited independently in schools in the UK and on a smartphone app. Data in Sample 1 were from 116 participants (48 male, 68 female), collected in groups of three to four participants in schools across Greater London, UK. We recruited participants in three age groups: 8–9 years (*N* = 30), 12–13 years (*N* = 43) and 16–17 years old (*N* = 43). All participants were fluent in English and did not have a history of neurological or psychiatric disorders. Participants were in schools in socially diverse areas of lower socioeconomic status to counteract the current recruitment bias towards youth with higher socioeconomic status (Fakkel et al., 2020). Unrelated data from these participants have been published elsewhere (Bowler et al., 2021; Dubois et al., 2022; Habicht et al., 2021; Moses-Payne et al., 2021).

Sample 2 included data from 75 participants (47 female, 26 male, 2 non-binary, fluid or undisclosed; mean age: 14.6 ± 0.24 years, range: 10–17 years) that was collected on the Brain Explorer smartphone app between 21 December 2020 and 23 July 2021. The Brain Explorer is a worldwide citizen science mobile application available from the Apple App Store and Android Play Store. People can complete behavioural tasks and psychiatric questionnaires on a smartphone or tablet (for further details, see https://brainexplorer.net/). Participants could choose to complete one questionnaire at a time and were not able to proceed until a response was provided for each item. Data were included from participants between 9 and 18 years old who had completed the AMI-CV questionnaire in their own time. There were no additional exclusion criteria or attention checks and participants were not reimbursed. This study was approved by the Research Ethics Committee of University College London (study numbers: 14261/001, 16711/002) and all participants provided informed consent. For participants younger than 16 years old, informed consent was also provided by their parent or legal guardian in both samples.

### Procedure

The aim of this study was to validate an apathy scale which is appropriate for children and adolescents, based on existing measurements for adults. Participants completed a questionnaire about feelings of apathy adapted for children from the adult version (Supplementary Table 1). The original, adult AMI is an 18-item self-report questionnaire which assesses apathy in terms of behavioural activation (tendency to self-initiate goal-directed behaviour), social motivation (level of engagement in social interactions) and emotional sensitivity (level of emotional engagement) using a five-point Likert scale (range 0–4; (Supplementary Table 1; Ang et al., 2017)). All items are reverse scored such that a positive score reflects greater apathy (lower motivation) in each domain. Scores are the mean rating of the items in each subscale and the total scale.

To develop the AMI-CV, we adapted items from the original adult version to increase their appropriateness for children and adolescents (Supplementary Table 1). We adapted the language of items or the sentence structure to reduce their complexity (items 2, 3, 5, 6, 9, 11, 12, 13, 14, 16, 18). For example, item 5: “I make decisions firmly and without hesitation” was adapted to “I make up my mind easily”. The language and reference timeline were simplified for item 8 (“I go out with friends on a weekly basis”, adapted to “I like to see my friends a lot”). An equal number of items were adjusted for the Behavioural Activation, Social Motivation and Emotional Sensitivity subscales (4 each). The remaining six items from the original questionnaire were unaltered from the adult version (AMI adult items 1, 4, 7, 10, 15, 17). The response options and scoring method remained the same as the original questionnaire for adults (Ang et al., 2017). The original and adapted AMI-CV questionnaire items in English, German and Russian are available in the online supplementary materials (Hewitt, 2022).

### Validation measures

To examine the construct and external validity of the AMI-CV, participants completed additional questionnaires. We reasoned that apathy in young people may be related to traits of both internalising and externalising disorders given the pervasiveness of apathy across several psychopathological dimensions and existing evidence in adults (Ang et al., 2017; Klar et al., 2022; Petitet et al., 2021). We used the Obsessive Compulsive Inventory–Child Version (OCI-CV; Foa et al., 2010) to assess traits related to Obsessive compulsive disorder, Mood and Feelings Questionnaire Short Child Self-Report (SMFQ-C; Messer et al., 1995) to assess depression traits, Screen for Child Anxiety Related Disorders (SCARED) short version (Birmaher et al., 1997) for anxiety traits and Barratt Impulsiveness Scale (BIS) child version (adapted from Patton et al., 1995) to assess trait impulsivity. The short form of the WASI-II including the Vocabulary and Matrix Reasoning subtests (Wechsler, 2011) was administered to estimate age-adjusted IQ and was conducted in Sample 1 (only). The older age group (16–17 years old, *N* = 43) in Sample 1 also completed the Apathy Evaluation Scale (AES; Marin et al., 1991), because the AES has only been validated in participants aged 16 or over. The construct validity of the AMI-CV questionnaire in the 16–17-year-old group was determined by calculating the correlation coefficients between the AMI-CV and AES. The order in which the tasks and questionnaires were administered was pseudo-randomised across participants.

### Data analysis

To determine the factor structure of the adapted questionnaire, we followed the procedure used to develop the AMI in adults (Ang et al., 2017) and the AMI caregiver version (Klar et al., 2022), and conducted exploratory factor analysis (EFA) on data from the two samples combined. We performed the Kaiser–Meyer–Olkin (Kaiser, 1974) test of sampling adequacy and Barlett’s test to determine that the AMI-CV items were sufficiently correlated for factor analysis to be appropriate. Next, we determined the number of factors using the modified procedure of Horn’s parallel analysis (Glorfeld, 1995; Horn, 1965). The modified procedure determines whether an eigenvalue is larger than what could be expected by chance. This reduces the tendency of the parallel analysis method to over-extract latent factors (Glorfeld, 1995). We conducted EFA (using maximum likelihood extraction), with Promax rotation, as we expected the factors to be correlated. EFA using the *Psych* and *Paran* packages in *R*.

Our aim was to identify a brief version of AMI which can be administered quickly to and easily understood by children as young as 8 years old. We followed the development of the AMI in adults and other questionnaires for assessing mental health traits in children (Birmaher et al., 1997; Foa et al., 2010) and removed items which did not robustly load onto a single factor according to best practice recommendations. As with the adult versions of this questionnaire, no particular item in the AMI-CV is necessary for the factor (i.e., items are not formative). This justifies item removal, which was also conducted in the adult version, because items in the AMI and AMI-CV are reflective of the underlying domains. We used the 40-30-20 rule (Howard, 2016) to determine items to retain in the adapted AMI-CV, which is an extended version of the criterion used in the adult version (Ang et al., 2017). This rule recommends that variables (1) load onto their primary factor above 0.40, (2) load onto alternative factors below 0.30, and (3) demonstrate a difference of 0.20 between their primary and alternative factor loadings and is consistent with recommendations by others (e.g., Guadagnoli & Velicer, 1988; Stevens, 2012). We also set additional criteria to remove items which were (4) multi-collinear (correlation coefficient among items > 0.8), and (5) low in communality (communality < 0.2; Child, 2006)).

After item reduction, we calculated Cronbach’s alpha for the total score and each subscale to determine the internal consistency of the AMI-CV. For the construct and external validity analysis, Shapiro–Wilk test was used to determine whether each variable was normally distributed, and Pearson or Spearman’s correlation was used. All materials, anonymised data and code for analysis are available for researchers on the Open Science Framework (Hewitt, 2022).

### Statistical power and sample size determination

The sample size in this study (*N* = 191) was sufficient for EFA based on recent recommendations (Mundfrom et al., 2005). We assumed low communality of items (due to the increased variability of children and adolescents) and a variable-to-factor ratio of 6 (the structure of the adult and caregiver AMI). This indicated a minimum of 160 participants (assuming a three-factor solution) in order to achieve excellent replicability (Maccallum et al., 1999; Mundfrom et al., 2005). To assess construct validity, the minimum sample size required to achieve 95% power was based on the effect sizes obtained by Ang et al., 2017 (total AES-AMI *r* = 0.6, alpha [two-tailed] = 0.05, required *n* = 30). For the external validity analysis, the minimum sample size to achieve 95% power was based on the correlation between the AMI-CV score and the measure of trait depression in Ang et al., 2017 (*r* = 0.26, alpha [two-tailed] = 0.05, minimum *n* = 187).

## Results

### Exploratory factor analysis (EFA)

Prior to conducting EFA, we determined whether sufficient correlations were present in the data for factor analysis to be appropriate using the Kaiser–Meyer–Olkin (KMO) test and Bartlett’s test of sphericity. The KMO test across all items of the AMI-CV suggested adequate sampling in both cohorts (KMO = 0.69). Bartlett’s test of sphericity also confirmed that correlations between the AMI-CV items were sufficient for factor analysis (χ^2^(153) = 761.21, *p <* 0.001). The modified Horn’s parallel analysis (Glorfeld, 1995; Horn, 1965) procedure revealed a three-factor solution replicating the adult and caregiver versions of the AMI.

Following the procedure for adult versions of the AMI, we conducted EFA with three factors and Promax rotation (maximum likelihood extraction), as we expected the factors to be correlated. The fit of this solution was assessed by two absolute fit indices: root mean square error of approximation (RMSEA) and standardised root mean square residual (SRMR). Both RMSEA and SRMR are indices of the difference between the observed and the hypothesised covariance matrices such that a lower value indicates a better model fit (Cangur & Ercan, 2015). We considered RMSEA < 0.08 and SRMR < 0.08 to be a good fit as in previous studies (Ang et al., 2017; Hu & Bentler, 1999; Klar et al., 2022). We also report Tucker–Lewis index (TLI), where a higher value indicated a better fit (Tucker & Lewis, 1973).

**Fig. 1:**
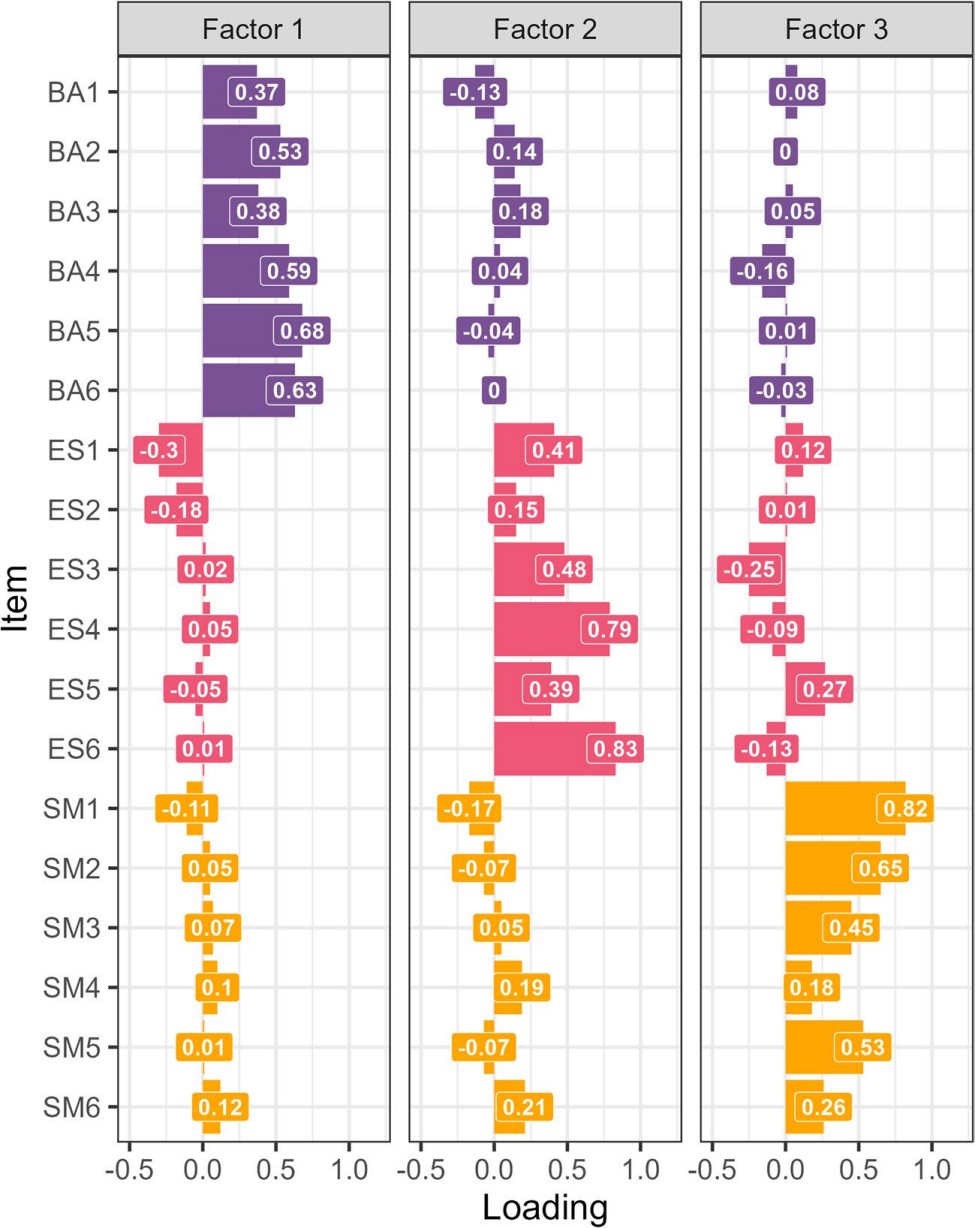
Factor loadings from the adapted questionnaire administered to participants in both samples (*N* = 191). The three-factor structure replicates the original subscales with similar model fit. Factor 1 predominantly features loadings from the behavioural subscale (BA: Behavioural Activation; purple). Loadings on factor 2 are predominantly from the emotional subscale (ES: Emotional Sensitivity; red), and loadings on factor 3 are mostly from the social subscale (SM: Social Motivation; orange)

The three-factor structure had similarly good fit to the data as reported in the adult and caregiver versions (RMSEA = 0.072, 90% CI = 0.057–0.086, SRMR = 0.06, TLI = 0.75). The AMI-CV items loaded on the three-factor subscales in a similar manner as in the original questionnaire (Fig. 1). Behavioural Activation (BA) items predominantly loaded onto factor 1 (mean absolute loadings: BA = 0.53, ES = 0.1, SM = 0.08). Emotional Sensitivity (ES) items mainly loaded on factor 2 (mean absolute loadings: BA = 0.08, ES = 0.51, SM = 0.13) and Social Motivation (SM) items mainly loaded on factor 3 (mean absolute loadings: BA = 0.06, ES = –0.15, SM = 0.48). The cumulative proportion of variance explained by these three factors was 0.31.

### AMI-CV item reduction

EFA revealed that the overall factor structure of the AMI-CV was similar to the originally retrieved factor structure in the adult AMI (Ang et al., 2017) and caregiver AMI (Klar et al., 2022). Six items (two from each subscale) did not load robustly onto a single factor according to our criteria (loadings < 0.4, signed loading difference between primary and secondary factors < 0.2, or communality < 0.2). Specifically, BA domain items (BA1: “I make up my mind easily” and BA3 “I don't like to laze around”), ES items (ES2: “After deciding something, I think about if I have made the wrong choice” and ES5: “I feel bad when I hear a friend has an accident or illness”), and SM items (SM4: “I often talk to other people without them talking to me first” and SM6: “I enjoy choosing what to do from a range of activities”). This suggested that these items do not reliably reflect a single construct across children and adolescents and were thus not useful to retain in the AMI-CV. We retained one item (ES1) which had a signed difference of 0.71 but an absolute difference < 0.2 because we expected the factors to be correlated and selected the Promax rotation method to allow this, based on data from the adult versions of the AMI. The inclusion/exclusion of this item did not affect any further results, including the number of factors estimated by Horn’s parallel analysis, construct validity of the AMI-CV compared with AES, external validity against related instruments and associations with age, IQ and gender, compared with an 11-item version with the item removed.

**Fig. 2:**
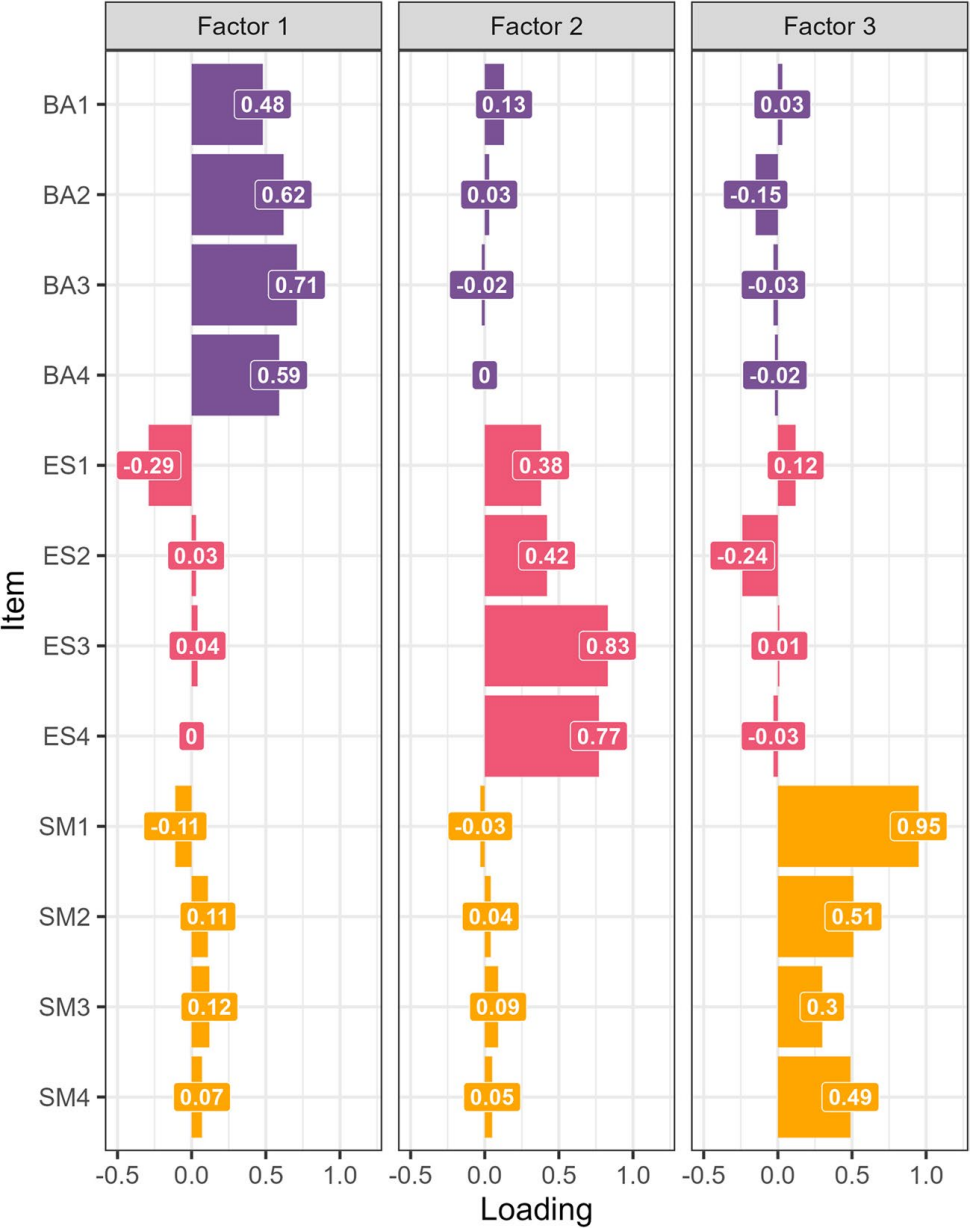
Factor loadings from the AMI-CV 12-item questionnaire across both cohorts (*N* = 191). The three-factor structure replicates the original subscales with similar model fit. Factor 1 predominantly features loadings from the behavioural subscale (BA: Behavioural Activation; purple). Loadings on factor 2 are predominantly from the emotional subscale (ES: Emotional Sensitivity; red), and loadings on factor 3 are mostly from the social subscale (SM: Social Motivation; orange). This suggests that we can capture three distinct domains of apathy with a more parsimonious, 12-item scale

As with the adult versions, the AMI-CV items were designed to be reflective of the underlying apathy dimensions. To simplify the measure for children, we removed these items (*n* = 6) and repeated the EFA with three factors and Promax rotation. The three-factor structure of the 12-item AMI-CV had a good fit to the data (RMSEA = 0.06, 90% CI = 0.029–0.086, SRMR = 0.05, TLI = 0.89), replicating the AMI in adults and the caregiver version. The cumulative proportion of variance explained by the three factors was 0.4 (factor 1 = 0.14, factor 2 = 0.13, factor 3 = 0.13), which is similar to related apathy scales (Klar et al., 2022; Radakovic & Abrahams, 2014) and similar brief scales of mood and feelings for children and adolescents (Messer et al., 1995)*.* Behavioural Activation (BA) items loaded exclusively on factor 1 (mean absolute loadings: BA = 0.6, ES = 0.09, SM = 0.1). Emotional Sensitivity (ES) items loaded positively onto factor 2 only (mean absolute loadings: BA = 0.05, ES = 0.6, SM = 0.05) and Social Motivation (SM) items loaded positively on factor 3 only (mean absolute loadings: BA = 0.06, ES = 0.1, SM = 0.56; Fig. 2). Scores from the BA and SM items correlated significantly (*r =* 0.19, *p =* 0.01), but ES scores did not significantly correlate with BA (*r =* 0.04, *p =* 0.61) or SM mean scores  (*r =* 0.07, *p =* 0.31). The range of factor loadings in the adapted AMI-CV reflects the ranges observed in several established instruments including the adult and caregiver versions (Ang et al., 2017; Klar et al., 2022) and related brief instruments for children, such as the widely used Short Mood and Feelings Questionnaire (sMFQ) (Messer et al., 1995). The AMI-CV captures the same domain-specific levels of apathy in children and adolescents and is directly comparable to the AMI in adults because the scores are calculated as mean values. BA scores reflect a child’s tendency to self-initiate goal-directed behaviours, SM scores index a child’s engagement with social interactions and ES probes an individual’s engagement with positive and negative feelings. We also conducted a cross-validated factor analysis which indicated the robustness of the three-factor solution and is detailed in the online supplementary material (Hewitt, 2022). Therefore, we continued the analysis with scores from 12-item AMI-CV.

### Internal consistency of the AMI-CV

We calculated the internal consistency of the AMI-CV 12-item version with Cronbach’s alpha. This was acceptable for the total score (α = 0.65) and similar for behavioural (α = 0.68), emotional (α = 0.67) and social subscales (α = 0.68). Cronbach’s alpha is determined by the average inter-item correlation and the number of items in a test, such that lower values might be expected in the short 12-item AMI-CV. To verify the internal consistency of the AMI-CV using an alternative method (not determined by the number of items), we conducted Spearman rank correlations for each item with their subscale (because subscales were not normally distributed according to Shapiro–Wilk *p* < 0.05); removing that item in the subscale calculation. We considered item–subscale average (for each domain) correlations of 0.4–0.5 to indicate acceptable internal reliability and the absence of redundant items (Briggs & Cheek, 1986; Clark & Watson, 1995), both of which are equally important in a brief scale. All individual item–subscale correlations were of moderate–large effect size (range: 0.29–0.56) and statistically significant after Bonferroni correction for multiple comparisons (Supplementary Table 3). The item–subscale correlations were stable for each AMI-CV subscale (mean ± SEM behavioural: 0.47 ± 0.03; emotional: 0.44 ± 0.06; social: 0.45 ± 0.05). This indicates that all AMI-CV items were moderately consistent with their subscales and no item was redundant.

### Construct validity of the AMI-CV

We assessed the construct validity of the 12-item AMI-CV in a group of older children (16–17 years; data not available for younger participants) from Sample 1 (*n* = 43) by comparing scores with the Apathy Evaluation Scale (AES; Marin et al., 1991). The AES consists of different subdomains to the AMI. Whereas the AES measures apathy in terms of behavioural, cognitive and emotional domains, the AMI measures behavioural, emotional and social domains. However, the total scores of both measures (across domains) would be expected to correlate to indicate construct validity. The AMI-CV total scores had excellent construct validity as they were strongly, positively correlated with AES total and subscale scores, indicating broad similarity between these measures (Table 1). The magnitude of the correlation coefficient (adult version: 0.61; child version: 0.63) for the AMI-CV total and AES total scores is a strong replication across these questionnaires in children and adults (Ang et al., 2017). The AMI-CV behavioural subscale also showed positive associations with all AES subscales. The AMI-CV ES did not significantly correlate with AES emotional (*rho =* 0.22, *p* = 0.15). However, this is to be expected as AES emotional subscale has only two items, and these address different conceptualisations of apathy to the AMI-CV ES. This finding reflects the adult version of this questionnaire in which the emotional subscale was only weakly correlated with the AES. AES emotional scale was however strongly correlated with the AMI-CV total score (as were all AES subscales) which indicates that the conceptualisations of apathy are broadly similar, replicating the adult version of this questionnaire (Ang et al., 2017). The AMI-CV SM subscales were typically not significantly associated with AES, which might be expected given the lack of social subdomain in the AES.

**Table 1:** Pearson correlations coefficients between Apathy-Motivation Index–Child Version 12–item (AMI-CV) and Apathy Evaluation Scale (AES) from *N* = 43 children aged 16–17 years

	AMI-CV total	AMI-CV-BA	AMI-CV ES ^b^	AMI-CV SM
AES total	**0.63 (*p*** **< 0.001)**	**0.68 (p < 0.001)**	0.06 (*p =* 0.69)	0.26 (*p* = 0.09)
AES behavioural	**0.41 (*p =*** **0.006)**	**0.67 (*p <*** **0.001)**	–0.03 (*p =* 0.84)	0.07 (*p =* 0.66)
AES cognitive	**0.59 (*p <*** **0.001)**	**0.53 (*p <*** **0.003)**	0.27 (*p =* 0.08)	0.28 (*p =* 0.07)
AES emotional ^b^	**0.51 (*p <*** **0.001)**	**0.39 (*p =*** **0.009)**	0.22 (*p =* 0.15)	0.28 (*p =* 0.07)
AES other ^b^	**0.36 (*p =*** **0.017)**	**0.64 (*p <*** **0.001)**	–0.17 (*p =* 0.27)	0.17 (*p =* 0.28)

AMI-CV scores are coded such that a higher score indicates greater apathy, whereas for AES a lower score indicates greater apathy (Marin, 1991). For ease of interpretation, the coefficient sign has been reversed to positive. BA: Behavioural Activation, ES: Emotional Sensitivity, SM: Social Motivation.

^b^ Spearman rank correlation

### External validity of AMI-CV

**Fig. 3:**
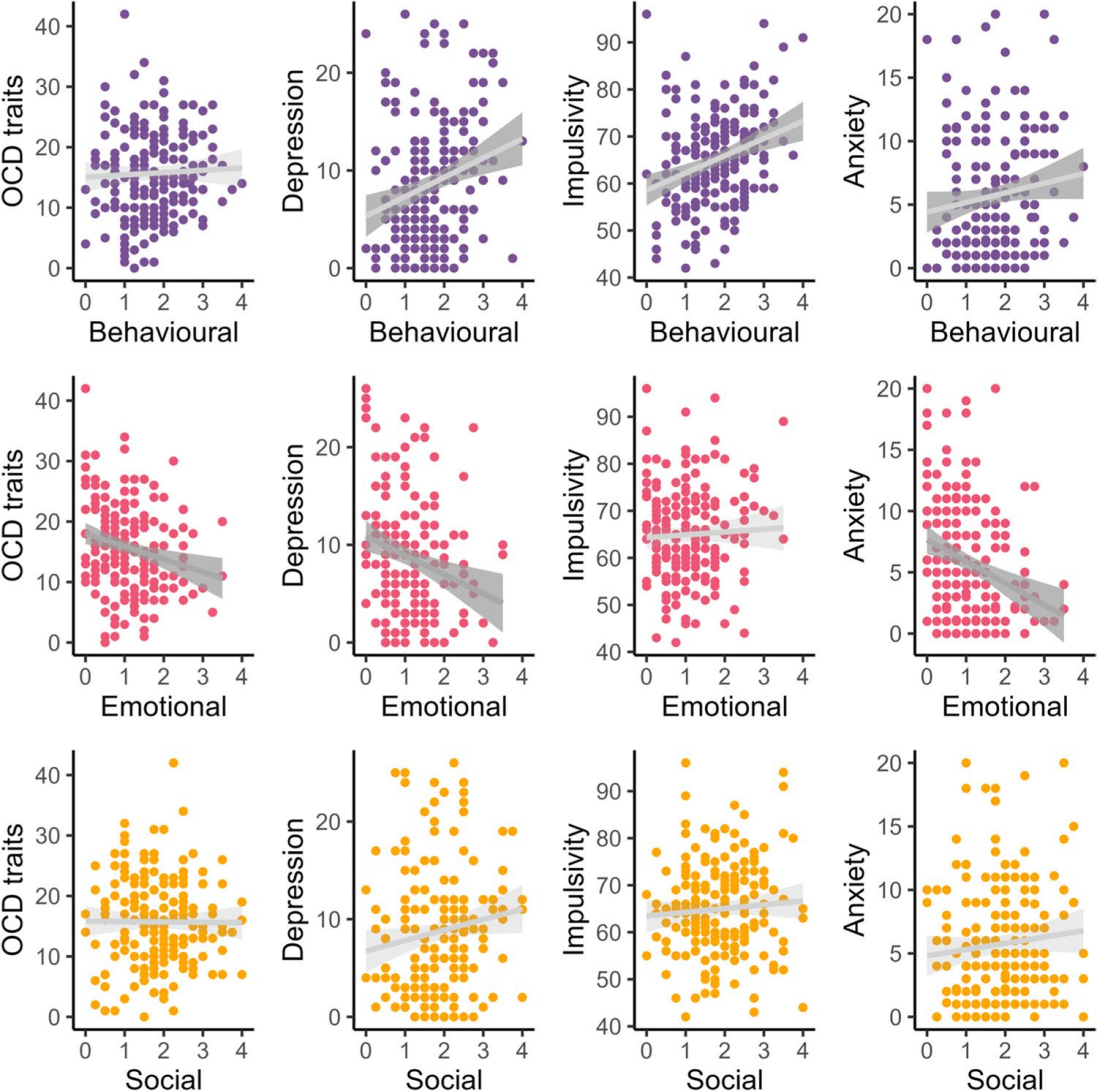
Scatter plots showing the relationship between AMI-CV 12-item scores and established measures of obsessive–compulsive, depression, impulsivity and anxiety traits in children. Greater AMI-CV score (*x*-axis) indicates greater apathy in that domain. Dark grey regression line indicates significant Spearman rank correlation (*p*_unc_ ≤ 0.004). Behavioural = behavioural activation subscale (purple), Social = social motivation subscale (orange), Emotional = Emotional sensitivity subscale (red), Impulsivity = Barratt Impulsivity Scale, Depression = Mood and Feelings Questionnaire Short Child Self-Report, OCD traits = Obsessive–Compulsive Inventory Child Version, Anxiety = Screen for Child Anxiety Related Disorders Short Version. Note that a small number of participants did not complete a specific questionnaire: Impulsivity (BIS-CV, *N* = 189), Anxiety (SCARED, *N* = 190), OCD traits (OCI-CV, *N* = 190), Depression (MFQ, *N* = 191)

To test the external validity of the AMI-CV, we examined the relationship between apathy scores and obsessive–compulsive, depression, anxiety and impulsivity traits as we expected apathy to be related to traits of internalising and externalising disorders (Fig. 3; Supplementary Table 2). Shapiro–Wilk tests revealed that Spearman rank correlations were appropriate. We report uncorrected *p* values but corrected significance level by the number of correlations using the Bonferroni method (α = 0.05 / 12), and so considered *p <* 0.004 to be statistically significant. This decreased the likelihood of false positive results.

#### Behavioural activation (BA)

The AMI-CV BA scores were significantly positively correlated with traits of impulsivity (BIS-CV total; *rho =* 0.29, *p*_unc_ < 0.001) and depression (SMFQ-C total; *rho =* 0.26, *p*_unc_ < 0.001). The magnitude and direction of the relationship between behavioural apathy and depression in children is a close replication of the adult and caregiver versions of this questionnaire (Ang et al., 2017; Klar et al., 2022). AMI-CV BA was not significantly associated with obsessive–compulsive (OCI-CV total; *rho =* 0.04, *p*_*unc*_ *=* 0.56) or anxiety traits (SCARED total; *rho =* 0.15, *p*_unc_ = 0.03) after correction for multiple comparisons.

#### Emotional sensitivity (ES)

The AMI-CV ES scores showed significant, negative correlations with depression (*rho* = –0.22, *p*_unc_ = 0.002), obsessive–compulsive (*rho* = –0.23, *p*_unc_ = 0.001) and anxiety traits (*rho* = –0.29, *p*_unc_ < 0.001). The distinct relationship between depression traits and behavioural compared with emotional apathy replicates the AMI in adults, using a different measure of depression traits (Ang et al., 2017). There was no relationship between AMI-CV ES and impulsivity (*rho* = 0.02, *p*_unc_ = 0.83).

#### Social motivation (SM)

We did not find any relationships between the AMI-CV SM subscale and impulsivity (*rho* = 0.06, *p*_unc_ = 0.39), anxiety (*rho* = 0.11, *p*_unc_ = 0.12) or obsessive–compulsive traits (*rho =* –0.04, *p*_unc_ = 0.63). The relationship between depression traits and AMI-CV SM was not significant after correction for multiple comparisons (*rho* = 0.17, *p*_unc_ = 0.02). However, the effect size is similar to the relationship between the AMI SM in adults and the Beck Depression Inventory (Ang et al., 2017).

### Apathy is stable across age, gender and age-adjusted IQ

We explored whether apathy probed using the AMI-CV was related to age, gender or IQ. We fit general linear models with dependent variables as AMI-CV scores and predictors as linear and quadratic effects of age, gender, and sample (school or online cohort) included as a covariate. To balance statistical power with control of type 1 error rate, we adjusted for multiple comparisons of these predictors (excluding intercepts) using Benjamini–Hochberg procedure to control the false discovery rate < 0.05 of this analysis and report corrected *p*-values (Benjamini & Hochberg, 1995). Two participants with gender identity other than male or female were excluded to preserve the assumption of homogeneity of variance.

Age was not significantly associated with AMI-CV BA (linear: β = 0.1, *p*_corr_ = 0.3; quadratic: β = 0.09, *p*_corr_ = 0.3), AMI-CV ES (linear: β = –0.08, *p*_corr_ = 0.3; quadratic: β = –0.03, *p*_corr_ = 0.67), or AMI-CV SM scores (linear: β = –0.14, *p*_corr_ = 0.12; quadratic: β = –0.03, *p*_corr_ = 0.67). Gender was also not significantly associated with apathy in any of the three domains: AMI-CV BA (male: β = –0.22, *p*_corr_ = 0.19), AMI-CV ES (male: β = 0.28, *p*_corr_ = 0.07) and AMI-CV SM (male: β = –0.17, *p*_corr_ = 0.3). The proportion of variance in AMI-CV BA (*R*^2^ = 0.04, *p =* 0.1) and AMI-CV ES (*R*^2^ = 0.05, *p =* 0.06) explained by these predictors was minimal and not statistically significant. However, AMI-CV SM scores were significantly increased in the online sample (β = 0.6, *p*_corr_ < 0.001; model *R*^2^ = 0.14, *p <* 0.001). The online data were collected during the COVID-19 pandemic (December 2020–July 2021), whereas the data in schools was collected prior to lockdowns in the UK which could explain the differences in social motivation across the cohorts. Age-adjusted IQ was assessed in Sample 1 only (*n* = 114), but this had no relationship with apathy in this cohort (AMI-CV total *rho* = 0.004, *p =* 0.97). In summary, the assessment of apathy using the AMI-CV was independent of age, gender and IQ.

## Discussion

We developed the AMI-CV, a brief questionnaire to measure levels of apathy in children and adolescents. This measure was derived from established adult versions (Ang et al., 2017; Klar et al., 2022). Across data from two samples, the adapted AMI-CV had similar psychometric properties to the adult versions, distinguishing three distinct subtypes of apathy in children aged 8–17 years old. Levels of apathy derived from the AMI-CV were not associated with IQ, age or gender. Apathy in children replicated relationships with internalising and externalising psychopathological traits found in adults.

We extended existing work using the AMI in adults and caregivers, by developing a developmentally appropriate apathy scale for children and adolescents. The adapted measure replicated the factor structure and the range of loadings in each domain of the adult versions across independent samples of children aged 8–17 years. Apathy domains had acceptable internal reliability and were robust after cross-validation. In a subgroup of older children, the AMI-CV showed excellent construct validity when assessed as total scores which correlated strongly with all domains of the Apathy Evaluation Scale and replicated the effect sizes seen in adults (Table 1). The AMI-CV is available in English with (unvalidated) suggested translations to German and Russian language for researchers in the online supplementary material (Hewitt, 2022). This measure for children can be directly compared with the adult versions to assess apathy across the lifespan.

A strength of the AMI-CV is its capacity to differentiate distinct apathy dimensions and their relationships with other mental health traits. We found positive relationships between behavioural apathy and depression and anxiety traits in children and adolescents (Fig. 3). Behavioural apathy reflects a child’s tendency for (reduced) goal-directed behaviour, which was also positively associated with trait depression in adults (Ang et al., 2017; Klar et al., 2022). This relationship is supported by the efficacy of behavioural activation therapy in major depressive disorder (Uphoff et al., 2020). In contrast, emotional apathy was negatively associated with depression and anxiety traits, which also replicates data in healthy adults (AMI) using a different measure of depression (Ang et al., 2017; Beck et al., 1996). The distinction between behavioural and emotional apathy extends to obsessive–compulsive traits which were negatively associated with emotional but not behavioural apathy. Finally, we also showed that behavioural apathy was positively related to impulsivity but emotional apathy was not. This replicates several large population samples of healthy adults (Petitet et al., 2021), and reveals that behavioural apathy and impulsivity are positively correlated in children as well. These findings indicate excellent external validity of the AMI-CV and provide further evidence to consider apathy as a multidimensional construct in its relation to other mental health traits, by extending this to children.

Notably, we did not find any associations between levels of social apathy and the other mental health traits which we measured in this study. This may be because social apathy scores were typically low (Fig. 3), and children and adolescents are particularly social (Ciranka & van den Bos, 2019; Jarman et al., 2021). However, the relationship between social apathy and depression traits that would be expected from data in adults would likely be significant with a larger sample and fewer statistical comparisons. It will be useful to compare scores of different apathy subcomponents across the lifespan, which the AMI-CV can facilitate, as evidence suggests that different subdomains of motivation also dissociate in healthy adult ageing (Cutler et al., 2021; Lockwood et al., 2021; Seaman et al., 2016).

Apathy probed using the AMI-CV was not associated with age, gender or IQ. This indicates that the AMI-CV can reliably assess distinct subtypes of apathy across different periods of childhood and adolescence. We also found that scores were generally similar across the online and school cohorts reported in this study, suggesting that the AMI-CV is suitable for online testing. However, social apathy was greater in the online cohort. There are two broad, possible explanations for this. Firstly, the online data collection coincided with the COVID-19 pandemic whereas data in schools was collected prior to lockdowns in the UK. Differences in the availability of social interactions could explain differences in social motivation of young people across the two samples. Alternatively, children and adolescents who spend more time online tend to have greater depression and anxiety (Carli et al., 2012; Lim & Nam, 2020), and the proportion of adults in online studies who report avoidance of social situations is significantly higher than in the general population (Shapiro et al., 2013). There may be differences in social motivation in general for young people that take part in studies online compared with in schools, which could be examined in future studies. Importantly, the factor structure of the AMI-CV was stable across both settings and similar to the adult versions of this scale.

Apathy and its inverse, motivation, are associated with important determinants of quality of life such as educational outcomes (Deci et al., 1991; Rosenzweig & Wigfield, 2016) and employment opportunities (Vansteenkiste et al., 2005). The putative impact of apathy on these may be mediated by its relationship with learning and decision-making. Future studies should investigate whether apathy in young people is associated with decision-making as has been shown in adults (e.g., Chong et al., 2016; Lockwood, Hamonet, et al., 2017b; Scholl et al., 2022) or with real-world behaviour (e.g., Dahl et al., 2018; Godefroy et al., 2021). The AMI-CV is a brief, psychometrically valid instrument to assess levels of apathy in young people which may have important implications for future life outcomes.

This study also has several limitations. The internal reliability of the subscales in the AMI-CV was acceptable but relatively low compared with the adult versions of this questionnaire. Subsequently, we showed that average item–subscale correlations were stable across subscales and within recommended ranges. Lower internal reliability is also in line with other studies showing weaker psychometric properties in young people (e.g., Austin & Huberty, 1993; Martínez-González et al., 2015) and suggests that in younger people the interpretation of concepts which relate to motivation may be more variable between individuals*.* Secondly, we have validated the AMI-CV in healthy children and adolescents from the general population. It is not yet clear whether the AMI-CV is suitable for the assessment of apathy in specific developmental or psychiatric disorders. Following the development of the adult versions, the AMI-CV was validated in terms of its convergent and divergent validity against several other psychopathological dimensions. However, some relevant dimensions (e.g., trait fatigue) were not covered due to a lack of age-appropriate measures and should be addressed in future studies. This study also did not include assessment of the test–retest reliability of the AMI-CV, which would provide insights into the temporal stability of the questionnaire. Future studies should assess this alongside the longitudinal development of apathy in young people over time. The sample size in this study was powered to detect the expected effects based on the data in adults and similar to the AMI-caregiver version (Klar et al., 2022). However, it was comparatively small for a psychometric validation study (due in part to the COVID-19 pandemic), so we encourage researchers to replicate these findings. Finally, we have provided two alternative language versions of the AMI-CV in German and Russian, which were translated by native speakers as suggestions for research use (online supplementary material, Hewitt, 2022), but these have not been specifically validated here.

In sum, the AMI-CV is a brief, valid measure of apathy in healthy young people. We show that it captures distinct levels of behavioural, social and emotional apathy in children replicating studies in adults. Future studies should investigate how differences in apathy during development are associated with behaviour, and how apathy across the lifespan is predictive of biopsychosocial outcomes.

## Declarations

### Open practices statement

The datasets and code used to analyse data in this study are available on the Open Science Framework (Hewitt, 2022). Paper versions of the AMI-CV in English, German and Russian are available in the online Supplementary Material (Hewitt, 2022).
